# Receptor for advanced glycation end products axis in critically ill patients

**DOI:** 10.1186/cc13401

**Published:** 2014-03-17

**Authors:** C Ingels, I Derese, PJ Wouters, G Van den Berghe, I Vanhorebeek

**Affiliations:** 1 KU Leuven, Belgium

## Introduction

Systemic inflammation caused by infection or trauma often leads to adverse outcome in critically ill patients. Binding of ligands to the receptor for advanced glycation end products (RAGE) activates several pathways, including the nuclear factor-kappa B pathway, which generates inflammatory cytokines, proteases and oxidative stress. RAGE activation has been suggested to link amplification and perpetuation of inflammation to subsequent organ damage and adverse outcome in sepsis, acute lung injury and myocardial dysfunction [1]. The soluble receptor, sRAGE, is thought to act as a decoy, thus protecting against further RAGE activation. High mobility group box 1 (HMGB1) is a nuclear protein that is released during cellular stress and damage. S100A12 is a neutrophil-derived protein that acts as a proinflammatory danger signal. Both are ligands for RAGE. We hypothesized that excessive RAGE activation is linked to adverse outcome in critically ill patients and that a different pattern of RAGE activation and inflammation may be present in patients according to underlying pathology.

## Methods

We measured sRAGE, HMGB1 and S100A12 serum levels upon admission, day 7 and the last day in the ICU in 405 critically ill surgical patients who needed intensive care for at least 7 days and in 69 matched healthy controls. We assessed the relation of these levels with clinical complications and outcome, in comparison with C-reactive protein (CRP) as a routinely measured clinical parameter of inflammation.

## Results

Upon ICU admission, levels of sRAGE, HMGB1, S100A12 and CRP were higher as compared with healthy levels. HMGB1, S100A12 and CRP remained elevated throughout the ICU stay but sRAGE decreased to levels lower than in healthy volunteers by day 7. sRAGE and CRP showed distinct time profiles during the ICU stay in patients undergoing cardiac versus other surgery and in patients with versus without sepsis upon admission. Elevated sRAGE upon admission, unlike CRP, was associated with need for renal replacement therapy, liver dysfunction, circulatory failure and mortality. Except for mortality, these associations remained in multivariate logistic regression analysis correcting for baseline risk factors.

## Conclusion

Critical illness alters several components of the RAGE axis. Elevated sRAGE levels upon admission to the ICU were associated with adverse outcome, independent of baseline pathology.
